# New geographical records for tick‐borne pathogens in ticks collected from cattle in Benin and Togo

**DOI:** 10.1002/vms3.1022

**Published:** 2022-12-12

**Authors:** Roland Eric Yessinou, Cristina Daniela Cazan, Luciana Cătălina Panait, Eyabana Mollong, Abel S. Biguezoton, Sarah Irène Bonnet, Souaïbou Farougou, Martin H. Groschup, Andrei Daniel Mihalca

**Affiliations:** ^1^ Communicable Disease Research Unit (URMaT) University of Abomey‐Calavi Cotonou Benin; ^2^ Department of Parasitology and Parasitic Diseases Faculty of Veterinary Medicine University of Agricultural Sciences and Veterinary Medicine of Cluj‐Napoca Cluj‐Napoca Romania; ^3^ CDS‐9, Molecular Biology and Veterinary Parasitology Unit Faculty of Veterinary Medicine University of Agricultural Sciences and Veterinary Medicine of Cluj‐Napoca Cluj‐Napoca Romania; ^4^ Laboratory of Applied Entomology Section: Agro‐Resources Protection, Faculty of Sciences University of Lome Lome Togo; ^5^ Unité Maladies à Vecteurs et Biodiversité (UMaVeB) Centre International de Recherche‐Développement sur l'Elevage en zone Subhumide (CIRDES) Bobo‐Dioulasso Burkina Faso; ^6^ Functional Genetics of Infectious Diseases Unit Institut Pasteur, CNRS UMR 2000 Université de Paris, Paris 7 France; ^7^ Animal Health Department Nouzilly France; ^8^ Friedrich‐Loeffler‐Insitut (FLI) Federal Research Institute for Animal Health Institute of Novel and Emerging Infectious Diseases Greifswald‐Insel Riems Germany

**Keywords:** bacteria, public health, Rickettsia aeschlimannii, tick, West Africa

## Abstract

**Background:**

Ticks are obligate hematophagous arthropods capable of transmitting a great variety of endemic and emerging pathogens causing diseases in animals and humans.

**Objectives:**

The aim of this study was to investigate the presence of *Bartonella* spp., *Rickettsia* spp.*, Borrelia burgdorferi* sensu lato (s.l.) and *Anaplasma phagocytophilum* in ticks collected from cattle in Benin and Togo.

**Methods:**

Overall, 396 (148 males, 205 females and 43 nymphs) ticks were collected from cattle in 17 districts (Benin and Togo) between 2019 and 2020. Ticks were pooled into groups of 2–6 ticks per pool according to individual host, location, species and developmental stage. The DNA of each pool was extracted for molecular screening.

**Results:**

PCR results revealed that 20 tick pools were positive for *Bartonella* spp. (Benin and Togo) and 23 tick pools positive for *Rickettsia* spp. (Benin), while all pools were negative for *A. phagocytophilum* and *B. burgdorferi* s.l. Sequence analysis of positive *Rickettsia* samples revealed the presence of *Rickettsia aeschlimannii*.

**Conclusions:**

The present study highlights the presence of zoonotic agents in ticks collected from cattle in Benin and Togo. This information will raise awareness of tick‐borne diseases among physicians and veterinarians, stimulate further studies to monitor these pathogens, and advise on necessary measures to control the spread of these zoonoses.

## Graphical abstract

A total of 396 ticks were collected from cattle between 2019 and 2020, and divided in pools (two to six ticks per pool) for molecular screening. The present study highlights the presence of zoonotic agents in ticks collected from cattle in Benin and Togo.

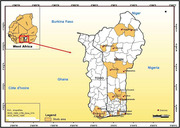

## 1 INTRODUCTION

Ticks are known to be important disease vectors, capable of transmitting different pathogens such as protozoa, bacteria, and viruses to a wide range of hosts, including humans (Dantas‐Torres et al., 2012). The distribution of tick‐borne pathogens is influenced by a series of climatic and environmental factors, as well as density and diversity of the host species (Ogden, 2017; Wikel, 2018). Among the tick‐borne pathogens, *Rickettsia, Anaplasma* or *Bartonella* species are of high importance in cattle (Kasaija et al., 2021). *Rickettsia* spp. are obligate intracellular alpha‐proteobacteria, with a worldwide distribution and transmitted to humans and animals by various arthropod vectors, including ticks (Parola et al., 2013). They have a high host adaptability, and the infections are important causes of morbidity and mortality worldwide (Diop et al., 2019; Saravanan et al., 2020). *Rickettsia* spp. are considered emerging zoonotic pathogens that are responsible for conditions such as the spotted fevers and typhus (Bhengsri et al., 2016). The continuous identification of new species or genotypes of *Rickettsia* (Parola et al., 2013; Portillo et al., 2015) in ticks brings up new questions on their true diversity, ecology and biology and the role of climate change in their distribution (Dantas‐Torres et al., 2012). This study provides information on these bacteria in West Africa, including Benin and Togo.

*Bartonella* spp. (Rhizobiales) are intracellular Gram‐negative bacteria. About 30 *Bartonella* species were described in a great variety of domestic and wild mammals worldwide (Kosoy et al., 2012). *Bartonella* spp. cause endocarditis in humans and domestic animals, including cattle (Erol et al., 2013). Detection of *Bartonella* spp. in bats was reported from Ghana and Nigeria (Billeter et al., 2012; Kamani et al., 2014). It has also been detected in rodents and their ectoparasites in Nigeria (Kamani et al., 2013). Although several *Bartonella* spp. were identified in ticks, their vectorial competence is still under discussion (Telford & Wormser, 2010). Despite the potential public and veterinary health importance, no studies were conducted on these bacteria in Benin and Togo.

*Borrelia burgdorferi* sensu lato (s.l.) complex comprises more than 20 genospecies and was described worldwide in a great variety of animals and vectors including ticks (Margos et al., 2019). *Borrelia burgdorferi* s.l. is one of the most important tick‐borne zoonotic agents in humans (Lefeuvre et al., 2020). They are maintained in nature by the interaction that exists with its vector ticks vertebrate reservoir hosts. *Borrelia burgdorferi* s.l. is transmitted by hard ticks and has been identified in Europe (Marchant et al., 2017), America (Scott et al., 2018) and Asia (Pukhovskaya et al., 2019), but few studies reported this zoonotic agent in Africa (Chitanga et al., 2014). While there are some reports of the presence of *B. burgdorferi* s.l. in Northern Africa (Elhelw et al., 2021), in Western Africa, mainly *B. crocidurae* and other species responsible for relapsing fever than Lyme borreliosis have been detected (Margos et al., 2019).

*Anaplasma phagocytophilum* (Anaplasmataceae) is an obligate intracellular bacterium known as a zoonotic tick‐borne pathogen and poses significant public health importance (Dumler & Walker, 2001). *Anaplasma phagocytophilum* was reported in several species of wild and domestic mammals worldwide, causing the human granulocytic anaplasmosis (HGA). In animals, it is responsible for reduction of milk production, abortion and also death (Stuen, 2007). *Anaplasma phagocytophilum* has been reported in various countries from Africa including Morocco, Zimbabwe, Tunisia, Algeria and South Africa (El Hamiani Khatat et al., 2021; Kelly et al., 2014; Nakayima et al., 2014). Currently, very few confirmed cases of *A. phagocytophilum* infection were reported in West Africa (Djiba et al., 2013).

In Africa, human tick‐borne diseases are underestimated and tick bites in humans go unreported due to the lack of awareness, knowledge of the risk of TBDs and the failure of the epidemiological surveillance system (N'koué Sambiéni et al., 2015; Rapp, 2014; Tutin, 2000). However, accurate identification of pathogens circulating between wild and domestic animals, ticks, and humans in a region is essential to facilitate diagnosis and treatment regimens, which depend on the pathogen involved. The lack of epidemiological knowledge on tick‐borne diseases induces confusion, with wrong diagnoses, and therefore, wrong treatment. The purpose of this study was to identify *Bartonella* spp.*, Rickettsia* spp*., B. burgdorferi* s.l. and *A. phagocytophilum* in ticks collected from cattle in Benin and Togo (West Africa).

## 2 MATERIALS AND METHODS

### 2.1 Study area and tick collection

**FIGURE 1:**
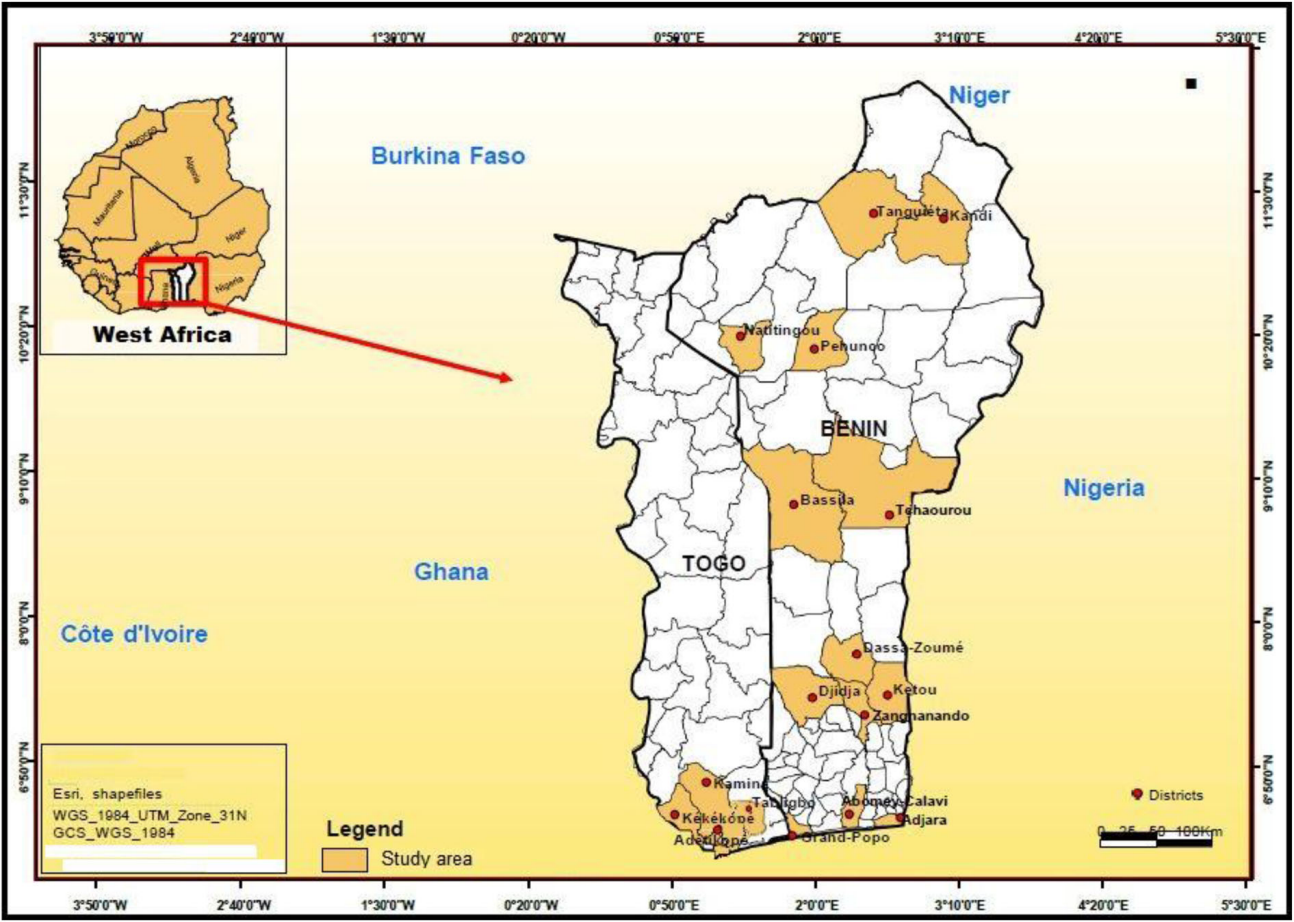
Geographical distribution of tick collection areas in Benin and Togo

A total of 396 ticks were collected from cattle in 2019–2020 in Benin and Togo (Figure 1). Ticks were preserved in 70% ethanol and transported to the Department of Parasitology and Parasitic Diseases of the University of Agricultural Sciences and Veterinary Medicine of Cluj‐Napoca. Identification of tick species was done under a stereomicroscope by using morphological keys (Estrada‐Peña et al., 2004; J. J. Walker et al., 2000; A. R. Walker et al., 2003).

### 2.2 Molecular assessment

Ticks were pooled into groups of 2–6 ticks per pool according to individual host, location, species and developmental stage. The DNA of each pool was extracted using ISOLATE II Genomic DNA Kit (Bioline Meridian Bioscience, Luckenwalde, Germany), according to the manufacturer's instructions and stored at −20°C.

Genomic DNA samples were screened for the presence of *Bartonella* spp*., Rickettsia* spp., *B. burgdorferi* s.l. and *A. phagocytophilum* by conventional and nested PCRs (Table 1), targeting the citrate synthase (*glt*A) gene of *Rickettsia* spp. and *Bartonella* spp., the flagellin B (*fla*B) gene of *B. burgdorferi* s.l. and the 16S rRNA gene of *A. phagocytophilum*.

**TABLE 1:** Primers and polymerase chain reaction conditions used for the detection of pathogens in ticks

			Temperature and duration of:					
Target species	Gene (∼amplicon length)	Forward and reverse primers (5'‐3') (Reference)	Initial denaturation	Denaturation	Annealing	Extension	Final extension	Number of cycles
*Bartonella* spp.	*glt*A (380–400 bp)	bart781: GGG GAC CAG CTC ATG GTG G bart1137: AAT GCA AAA AGA ACA GTA AAC A (Norman et al., 1995)	95°C, 5 m	95°C, 30 s	52°C, 30 s	72°C, 30 s	72°C, 5 m	35
*Rickettsia* spp.	*glt*A (381 bp)	Rsfg877: GGG GGC CTG CTC ACG GCG G Rsfg1258: ATT GCA AAA AGT ACA GTG AAC A (Regnery et al., 1991)	95°C, 2 m	95°C, 30 s	58°C, 30 s	72°C, 30 s	72°C, 2 m	35
*B. burgdorferi* s.l. (nested PCR)	*flaB* (650 bp)	FlaLL:ACA TAT TCA GAT GCA GAC AGA GGT FlaRL: TGT TAG ACG TTA CCG ATA CTA ACG (Barbour et al., 1996)	95°C, 5 m	95°C, 30 s	55°C, 30 s	72°C, 45 s	72°C, 5 m	35
	*flaB* (350–400 bp)	FlaLS: AAC AGC TGA AGA GCT TGG AAT G FlaRS: CGA TAA TCT TAC TAT TCA CTA GTT TC (Clark et al., 2013)	95°C, 5 m	95°C, 30 s	59°C, 30 s	72°C, 30 s	72°C, 5 m	35
*A. phagocytophilum* (nested PCR)	16S rRNA (945 bp)	Ge3a: CAC ATG CAA GTC GAA CGG ATT ATT C Ge10r: TTC CGT TAA GAA GGA TCT AAT CTC C (Massung et al., 1998)	95°C, 2 m	94°C, 30 s	60°C, 30 s	72°C, 1 m	72°C, 5 m	40
	16S rRNA (570 bp)	Ge9f: AAC GGA TTA TTC TTT ATA GCT TGC T Ge2: GGC AGT ATT AAA AGC AGC TCC AGG (Massung et al., 1998)	95°C, 2 m	94°C, 30 s	55°C, 30 s	72°C, 1 m	72°C, 5 m	30

Abbreviations: m, minute; s, second.

The PCR amplifications were performed in 25 μl reaction volume, containing 12.5μ1 Green PCR Mastermix (Rovalab GmBH, Teltow, Germany), 6.5 μl of ultra‐pure water, 1 μl (10 pmol/μl) of each primer (Table 1), and 4 μl of isolated DNA aliquot. One negative control (ultra‐pure water) as well as a positive control were included. For the nested PCR targeting the *flaB* gene of *B. burgdorferi* s.l. and 16S gene of *A. phagocytophilum*, a second reaction was performed in 25 μl reaction volume, containing 12.5μ1 Green PCR Mastermix (Rovalab GmBH), 9.5 μl of ultra‐pure water, 1 μl (10 pmol/μl) of each of the two primers (Table 1), and 1 μl aliquot of the first PCR reaction product.

The PCR was performed using the T1000™ Thermal Cycler (Bio‐Rad, London, UK). Amplification products were visualized by electrophoresis on 1.5% agarose gel stained with ECO Safe 20,000 × Nucleic Acid Staining Solution (Pacific Image Electronics, New Taipei, Taiwan) and their molecular weight was assessed by comparison to a molecular marker (O'GeneRuler™ 100 bp DNA Ladder, Thermo Fisher Scientific Inc., Waltham, MA, USA). PCR products were purified using the ISOLATE II PCR and Gel Kit (Bioline Meridian Bioscience) and sent for sequencing (Macrogen Europe, Amsterdam, the Netherlands).

### 2.3 Sequencing

All sequences were analyzed and edited using Geneious® 4.85 software. Basic Local Alignments Tool (BLAST) analyses (https://blast.ncbi.nlm.nih.gov) were conducted to compare all the obtained sequences with the ones deposited in the GenBank™ database.

## 3 RESULTS

### 3.1 Molecular detection

A total of 396 (148 males, 205 females and 43 nymphs) ticks were collected from cattle in 17 districts (Benin, Togo) (Figure 1) and divided into 96 pools. Overall, PCR results showed that 20 tick pools were positive for *Bartonella* spp. and 23 pools for *Rickettsia* spp.

In Benin, pools were positive for *Bartonella* spp. (25.39%) and *Rickettsia* spp. (30.15%). *Bartonella* spp. and *Rickettsia* spp. have been detected in *Amblyomma variegatum, Hyalomma rufipes* and *Rhipicephalus microplus* (Table 3). The PCR targeting the *glt*A gene allowed identifying *Bartonella* spp. and *Rickettsia* spp. in several districts in Benin, such as Abomey‐Calavi, Bassila Dassa‐Zoumè, Grand‐Popo, Kétou, Natitingou, Pehunco, Tchaourou and Zangnanando (Table 2).

**TABLE 2:** *Bartonella* spp. and *Rickettsia* spp. detected in different study sites

			*Bartonella* spp.			*Rickettsia* spp.		
Country	District	Pools (n)	M	F	N	M	F	N
Benin	Abomey‐Calavi	6 (26)	‐	1/6	‐	1/6	1/6	‐
	Adjara	4 (19)	‐	‐	‐	‐	‐	‐
	Bassila	3 (8)	‐	‐	‐	‐	1/3	‐
	Dassa‐Zoumè	7 (36)	1/7	2/7	‐	1/7	1/7	‐
	Djidja	4 (21)	‐	‐	‐	‐	‐	‐
	Grand‐Popo	6 (20)	1/6	1/6	‐	‐	1/6	‐
	Kandi	4 (19)	‐	‐	‐	‐	‐	‐
	Kétou	3 (18)	‐	‐	‐	1/3	1/3	‐
	Natitingou	4 (23)	1/4	2/4	‐	1/4	2/4	‐
	Pehunco	4 (19)	1/4	1/3	‐	1/4	3/4	‐
	Tanguiéta	5 (20)	‐	‐	‐	‐	‐	‐
	Tchaourou	6 (32)	‐	1/6	‐	1/6		‐
	Zangnanando	7 (30)	1/7	1/7	‐	2/7	1/7	‐
	Subtotal	63 (291)	5/63	9/63	‐	8/63	11/63	
Togo	Kamina	10 (27)	‐	1/10	‐	‐	1/1‐	‐
	Kékékopé	6 (23)	‐	‐	‐	‐	1/6	‐
	Adétikopé	9 (25)	‐	‐	‐		‐	‐
	Tabligbo	8 (30)	3/8	‐	‐	‐	2/8	‐
	Subtotal	33 (105)	3/33	1/33	‐	‐	4/33	‐
Total	96/396	8/96	10/96	‐	8/96	15/96	‐	

In Togo, *glt*A rickettsial DNA was detected in four out of 33 tested tick pools, including *A. variegatum*, *R. microplus*, *Rhipicephalus muhsamae*, and *Rhipicephalus sulcatus* pools. The DNA of *Rickettsia* spp. was identified in *A. variegatum*, *Hyalomma truncatum*, *Rhipicephalus annulatus* and *R. microplus* (Table 3). *Bartonella* spp. were identified in Kamina and Tabligbo, while *Rickettsia* spp. were found in Kamina, Kékékopé and Tabligbo. All samples tested for *B. burgdorferi* s.l. and *A. phagocytophilum* in this study were negative.

**TABLE 3:** *Bartonella* spp. and *Rickettsia* spp. detected in tick species

		Number of tested ticks				PCR positive pools	
Country	Tick species	Male	Female	Nymph	Pools (*n*)	*Bartonella* spp.	*Rickettsia* spp.
Benin	*Amblyomma variegatum*	73	62	12	29 (147)	24.13 7/29	48.27 14/29
	*Hyalomma rufipes*	13	8	‐	9 (21)	33.33 3/9	33.33 3/9
	*Rhipicephalus microplus*	15	77	31	25 (123)	24 6/25	8 2/25
	Subtotal	101	147	43	63 (291)	25.39 16/63	30.15 19/63
Togo	*Amblyomma variegatum*	24	16	‐	8 (40)	12.5 1/8	12.5 1/8
	*Hyalomma truncatum*	6	5	‐	7 (11)	‐	14.28 1/7
	*Rhipicephalus annulatus*	2	6	‐	4 (5)	‐	25 1/4
	*Rhipicephalus decoloratus*	‐	3	‐	1 (3)	‐	‐
	*Rhipicephalus lunulatus*	‐	3	‐	2 (3)	‐	‐
	*Rhipicephalus microplus*	4	21	‐	5 (26)	20 1/5	20 1/5
	*Rhipicephalus muhsamae*	‐	2	‐	1 (2)	100 1/1	‐
	*Rhipicephalus sanguineus*	8	2	‐	4 (10)	‐	‐
	*Rhipicephalus sulcatus*	3	‐	‐	1 (3)	100 1/1	‐
	Subtotal	47	58	0	33 (105)	12.12 4/33	12.12 4/33
Total						20.83 20/96	23.95 23/96

### 3.2 Sequencing

The *glt*A gene sequences carried out from *Bartonella* spp. obtained from two pools of *R. microplus* in Benin were closely related to a *Bartonella* strain from China (98.23% and 90.76% identity) (accession No. KX354203). Analysis of the *glt*A gene from *Rickettsia* spp. revealed of sequences which had 97.29%–100% identity to various *R* *ickettsia aeschlimannii* sequences: accession No. MH267736, MH932014, MH932013; MH675642‐MH675648, MK608659‐MK608560; KJ663742; KY233219; KU961540 obtained from China, France, Italy, Lebanon and Crimean Peninsula. *Rickettsia aeschlimannii* was detected in seven tick pools including *A. variegatum*, *R. microplus* and *H. rufipes* collected in Benin.

## 4 DISCUSSION

Data on the distribution of tick‐borne pathogens are necessary for designing appropriate control measures and future approaches to more comprehensive surveillance (Gondard et al., 2017). Very few data exist on this topic in West Africa where a high diversity of tick species was recorded in Benin and Togo (Yessinou et al., 2022). Their presence and distribution can be explained by ecological and climatic conditions that are favourable or unfavourable for the development of ticks in certain countries. All these ticks identified in the study area are capable of playing a role in the transmission and/or maintenance of *Bartonella* spp. and *Rickettsia* spp. (Dantas‐Torres et al., 2012). In this study, five tick species collected from cattle were positive for the presence of *Bartonella* DNA, namely *A. variegatum, R. microplus, H. rufipes, R. muhsamae*, and *R sulcatus*. Several species of Bartonella have been reported in ticks, mammals and humans so far (Tsai et al., 2010; Vayssier‐Taussat et al., 2015). Our findings represent the first detection of *Bartonella* spp. in hard ticks in Benin and Togo. *Bartonella* spp. were previously also detected in rodents and ticks from Nigeria (Kamani et al., 2013).

Sequencing results also confirmed that *A. variegatum, R. microplus* and *H. ruffipes* were infected with *R. aeschlimannii*, a tick‐borne pathogen, reported in 1997 in *Hyalomma marginatum* ticks collected in Morocco (Beati et al., 1997). For the first time, the presence of *R. aeschlimannii* in ticks collected from cattle in Benin was shown in this study. In Senegal, Mediannikov et al. (2010) reported *R. aeschlimannii* in *H. (m) rufipes, H. truncatum* and *Rhipicephalus evertsi evertsi* ticks collected from cows, donkeys, sheep, goats and horses. Others studies in Niger, Mali, Nigeria Ivory‐Coast and Burkina‐Faso identified *R. aeschlimannii* in *Hyalomma* spp. (Ehounoud et al., 2016; Kamani et al., 2015; Parola et al., 2001; Tomassone et al., 2016), but also in Europe (Fernández Soto et al., 2003; Punda‐Polic et al., 2003). *H*yalomma *marginatum* appears to serve as a vector but also as a reservoir of *R. aeschlimannii* (Parola et al., 2005). *Rickettsia aeschlimannii* was reported in other tick genera including *Amblyomma, Dermacentor, Ixodes* and *Rhipicephalus* (Orkun et al., 2014a; Rumer et al., 2011; Toma et al., 2014). This pathogen is implicated in several cases of fever in humans but also reported in several places in animals (Orkun et al., 2014b; Tosoni et al., 2016). The symptoms following an infection are similar to those of Mediterranean spotted fever caused by *Rickettsia conorii* (Rovery & Raoult, 2008). Raoult et al. (2002) reported cases of human infection caused by *R. aeschlimannii* in France.

New tick‐borne pathogens infecting humans have increased in recent years, thus playing an important role in public health. In sub‐Saharan Africa, *R. aeschlimannii* is known as a potentially important pathogen and was identified in humans, animals and ticks (Parola et al., 2013). In these regions, febrile illnesses in breeders, farmers and animal health professionals may be caused by rickettsiosis (Moumouni et al., 2016). Mediannikov et al. (2010) showed that tick‐borne rickettsioses are among the causes of acute nonmalarial febrile diseases. Traditionally, in West Africa, almost all febrile conditions are generally considered to be linked to malaria and are treated as such without further investigations, but other tick‐borne pathogens might be involved. Thus, clinical manifestations of Rickettsioses should be considered in the differential diagnosis of the malaria in patients mainly in rural areas or locations of ruminants rearing.

It should also be noted that the identification of pathogen DNA in a tick does not imply that it is necessarily a biological vector. Studies must be conducted to prove the vector competence of the tick species. The growth of the world population, the transformation of natural habitats, the global changes and the practices of use of the fauna are some of the factors that modify and facilitate the interactions between wild and anthropic environments (Cable et al., 2017). These growing contacts between wildlife and domestic animals, humans and ticks gradually promote the exchange of pathogens that can have harmful health consequences on the animals and humans.

## 5 CONCLUSION

This study reports the identification and distribution of pathogens in hard ticks collected from cattle in West Africa. These results suggest the need to include bartonellosis and rickettsiosis among the causes of febrile illnesses among breeders, para‐veterinarians, veterinarians and travellers in West Africa. Doctors should also consider these tick‐borne illnesses as a differential diagnostic with malaria. On the other hand, the role of domestic and wild animals in the epidemiology of diseases transmitted by ticks requires further investigation.

## AUTHOR CONTRIBUTIONS

*Conceptualization, data curation, formal analysis, investigation, methodology, and writing—original draft*: Roland Eric Yessinou. *Conceptualization, formal analysis, and methodology*: Cristina Daniela Cazan. *Conceptualization, formal analysis, and methodology*: Luciana Cătălina Panait. *Investigation*: Eyabana Mollong. *Conceptualization and methodology*: Abel S. Biguezoton. *Conceptualization, methodology, and writing—original draft*: Sarah Irène Bonnet. *Conceptualization*: Souaïbou Farougou. *Conceptualization, Data curation, Methodology*: Martin H. Groschup. *Funding acquisition, Supervision, Writing – review & editing*: Andrei Daniel Mihalca.

[Correction added on 21 December 2022, after first online publication: The Author contributions section was updated.]

## CONFLICT OF INTEREST

The authors declare no conflict of interest.

### ETHICS STATEMENT

No ethical approval was required, as this study does not involve clinical trials or experimental procedures. The cattle's are still alive and used for milk and meat production. This study did not involve endangered or protected species.

### PEER REVIEW

The peer review history for this article is available at https://publons.com/publon/10.1002/vms3.1022.

## Data Availability

The data that support the findings of this study are available from the corresponding author upon reasonable request.
